# Galvanic Experiments, Applied to the Uses of Medicine

**Published:** 1802-12-01

**Authors:** 

**Affiliations:** Stockholm


### *Galvanic Experiments, applied to the Uſes of Medicine; by Dr.* QUENSEL, *of Stockholm.*

THE Galvanic pile of Volta, of which I have made uſe, conſiſted of thirty pieces of ſilver, and as many of zinc, about the ſize of a Swediſh dollar, (equal to half a crown Engliſh) with pieces of cloth that were dipped in a ſaturated ſolution of common ſalt. This pile was kept in its ſituation by two perpendicular tubes of glaſs, and inſulated by a plate of glaſs laid under it. I have ſometimes brought into connexion with the former another pile, compoſed of fifty pieces of copper, and as many of zinc, of a ſimilar form. The manner of conducting the Galvanic ſtimulus to the affected parts, was by means of gilt and ſilver wires paſſing through glaſs tubes, and ending in knobs. The order in which I conſtructed the pile was as as follows: Undermoſt zinc, then wet cloth, ſilver, zinc, and ſo on. In caſes of deafneſs, I have always applied the zinc wire to the ear, while the ſilver wire was hanging down into a baſon with ſalt water; if it was not likewiſe applied to the patient, viz. to the tuba Euſtachii or to the other ear. I generally found, that it was leſs painful, if the Galvanic chain had been ſhut with the fingers in a baſon of ſalt water, than if the oppoſite pole was touched by means of a piece of metal held in the hand, which was previouſly moiſtened with ſalt water. In affections of the organ of hearing, I have never employed above twenty pairs of plates; and, ſometimes, if the patient was very ſenſible, I have been obliged to diminiſh them to fifteen and twelve. In more than two hundred perſons, on whom I have made experiments, I obſerved that the action of Galvaniſm varies not only in different people, but in the ſame perſon at different times. On the whole it may be ſaid to increaſe the animal heat, as almoſt all the perſons on whom I tried the Galvanic experiments, felt themſelves warmer, and their perſpiration to be conſiderably increaſed. Thoſe who had only perſpired the firſt day in the face, felt a general ſweat the following day, while the topical ſweat in the face had diminiſhed. In ſome, the ſweat enſued the night after the application.

Galvaniſm, particularly at the zinc pole, attracts the blood to the place where it is applied, and I have often found blood in the ears of the patients ſome time after the experiments, and in one caſe it trickled down the cheeks. A young lady, who had a paralyſis of the tongue, and a difficulty of ſpeech, derived, from the application of Galvaniſm, blue ſuggilated ſpots on the ſkin. Another had the head ach, and his noſe began to bleed, on having applied the zinc pole to the palate, while he held the chain of the other pole in his hand. Sometimes ſmall wounds were occaſioned in the ears by the application of Galvaniſm, and I remember, in one or two inſtances, of a ſlight delirium, with a head ach, and an inclination to ſleep, which laſted for about 24 hours, and ſeemed to be removed by a foot bath. Some patients perceived, during the application, a particular ſound, as from a ſhot, or ſometimes the ſound of trumpets, &c. but I never could take theſe phenomena for favourable ſymptoms; and in general, I have not been happy enough to obſerve ſuch ſpeedy and favourable effects in caſes of deafneſs, as have been announced by other phyſicians in ſundry German newſpapers. Some perſons perceived a particular ſulphurous or metallic taſte, whenever the poſitive wire was introduced into the ear, while the other was held in the moiſt hand. A patient of a ſtrong conſtitution, having one day uſed the Galvaniſm longer than than usual, had a diarrhœa soon after, and his bowels were always moved during the continued application of Galvanism. The same symptoms I observed in a man who had applied the experiments to his arms.

Case I. In October, last year, I tried Galvanism on a girl, six years of age, who, in her second year, (in consequence of a scarlet fever,) had lost the power of hearing and of speech to such a degree, that she could not hear the noise of a cannon shot, even if she stood close by. From the ears issued a thin pus of a bad quality, and the child was, on the whole, in a bad state of health, and seemed to have much disposition to rickets; she was extremely emaciated, and had frequent diarrhœas, &c. On having applied Galvanism to the ears for a few days, the purulent matter ceased to run, and on the 6th of November the ears became sore and very sensible. Two days after she began to perceive strong sounds; the running of the ears appeared again, but the diarrhœa had diminished. On the 11th, I observed that ear-wax had been secreted in the ear which was galvanised, and the matter had received a greater consistency; the sensibility of the ear was, at the same time, considerably increased. As the left ear, which I had hitherto galvanised, began to bleed, I applied Galvanism to the right ear, and afterwards I galvinised both ears alternately. The hearing had so considerably increased, that she heard the ringing of the bells, the voice of the birds, and her own name, when called by it, which indeed was the only articulated sound she could understand. On the whole, her state of health had been much improved during the application of Galvanism, the diarrhœa had entirely ceased, her appetite was much better, &c. all which I considered as owing to the continued use of Galvanism, no other medicines having been given. It yet remains to be seen, whether the continued application of Galvanism will perform a radical cure of deafness in this patient, or only act as a palliative; at least, a decrease of hearing was perceived whenever she had discontinued the Galvanism only one day.

Case II. A man, 36 years of age, who was deaf and dumb from his earliest infancy, used the Galvanic agents in the same manner as is related in the foregoing case; but though he daily continued its application for two months together, he perceives no alteration, except an increased perspiration, and some congestions of blood towards the head, in consequence of which he becomes giddy, but is as deaf as he was before he began the experiment, though he sometimes fancies he can hear a little better.

Case III. A man, aged 23 years, who, in his third year, had become deaf and dumb in consequence of a sudden fright, was galvanised galvanifed on the 2d of November. On November 13, he gave figns that he could hear ftrong founds; and fome days after, I obferved fome ear wax which had been fecreted in the ear; this we proceeded to galvanife; his hearing, however, is fo very little improved, that he cannot hear the penetrating found of a French horn, though, it fhould feem, that he hears the found of two books when clapped together, or of fingle words very loudly announced; which laft fact, however, cannot be ftrictly afcertained, as it is known that deaf people perceive the meaning of words from the change in the mufcles of the face of the fpeaker, and not by their being heard.

In this manner, I have applied the Galvanic pile in a great number of cafes of deafnefs; but on the whole, the patients relapfed into their former ftate, though they had fancied that the hearing was improved during the actual application. In feveral cafes, however, the ear wax began to be fecreted by means of the Galvanifm, or to affume a better quality.

Case IV. A man of middle age, having, during eight days, fuffered a violent otalgia, attended with running of the ears, had recourfe to the Galvanic chains three times within eight days. After it had been applied the firft time for about ten minutes, the pains confiderably diminifhed, and he flept tolerably well; the following night, after the fecond experiment, the pains decreafed ftill more, and the third experiment made them entirely difappear.

In head-achs, particularly if they were produced by a rheumatic caufe, Galvanifm proved of the greateft fervice, and gave almoft inftant relief, when the pains had lafted for feveral months. In this cafe I applied the wire of the zinc pole to the temples, or the forehead; or I ordered it to be held in the mouth, while the other was taken in the hand.

Case V. A married lady, thirty years of age, during a month, had a pain in the face, very much refembling the Fothergillian face-ach, (*Tic Douloureux*) which always arofe from a fpot at the proceffus orbitalis offis zygomatici, thence fpreaditfelf like a line from a centre, in all directions of the face, and making but fhort remiffions. The hæmicrania, and the pains in the teeth and jaws were at the fame time almoft infupportable. Concerning this painful complaint, fhe had confulted the moft refpectable phyficians, and at laft fhe was ordered to have a mercurial ointment rubbed in at the affected place, and to take pills of cicuta, by which fhe feemed to be fomewhat relieved, but the pains recurred from time to time with increafed violence. No other caufe could be affigned for this affection but a retrograde gout, the patient having bathed her feet at the time when fhe was affected with the gout, after which which a hæmicrania had inftantly enfued; by proper remedies, however, the gout appeared again, and the head-ach ceafed. The patient began now to drink mineral water, by which fhe likewife got rid of the gout; but having one day caught cold, the above-mentioned violent pain in the face returned. After having difcontinued all other medicines, I prepared a pile of fifteen ftrata, and applied the wire of the zinc pole to the fpot whence the pain arofe, and fometimes to the forehead, the temples, and the lower jaw, while the Galvanic chain was fhut by the hand, with which fparks were continually drawn. The natural heat of the patient increafed, but no fweat enfued. After having, in this manner, made three experiments the firft day, the pains abated in a confiderable degree. Galvanifm being thus continued, during eight days, twice a day, the painful affection ceafed entirely, without having appeared again.

CASE VI. A girl of twelve years of age, had, in her third year, the fmall-pox, the eruption of which had not been quite perfect: fince that time fhe had loft the power of fpeech, was difordered in her mind; and though fhe was fometimes better, fhe always remained in the ftate of an ideot; but particularly fhe feemed to be entirely deprived of memory. Although fhe could move the tongue and lips without any impediment, fhe only pronounced few words, and at the fame time very unintelligible. After having applied Galvanifm to the neck, and the inner parrts of the mouth, the root of the tongue, &c. no other change was obferved in the fpace of five weeks, but that the child had become more filly and fretful, and that a tooth-ach, which fhe frequently fuffered, had entirely difappeared; blue fuggilated fpots were obferved on the neck, where Galvanifm had been applied.

CASE VII. A man, twenty-four years of age, had, feveral years ago, been cured of an hydrocele by means of cauterization, after which a tumour of the tefticles remained. I applied the wire of the zinc pole to the fpermatic veffels, at the upper end of one of the tefticles, while the oppofite fide of the fcrotum was touched with the wire of the filver pole. After having ufed Galvanifm for about three days, the tumour feemed to have diminifhed and become fofter; but the patient found it fo troublefome as not to be able to continue the experiment.

---

The medical application of Galvanifm is become quite fafhionable among the practitioners of Germany; and all the public papers magnify the great advantages which have been obferved to refult from it in cafes of deafnefs and blindnefs, and other complaints in which the nervous fyftem is chiefly affected. We have fome reafon to fear that many of thefe reports are, in a great meafure, exaggerated; a circumftance by which the powerful Galvanic agent is is likely to be difcredited in the eyes of the public, and thus; to fhare the fate which the medical ufe of Electricity has of late experienced. As the medical art, however, may juftly hope to reap much benefit from the medical application of that ftimulus, it is our fincere wifh, that it may be put to a fair trial by intelligent and impartial obfervers, on whofe ftatements we can rely with fecurity; and whatever may be publifhed relative to the medical application of Galvanifm, we fhall faithfully lay before our readers, as a fubject which feems to engage the attention of all ranks of people, and which has a particular claim to be recorded in this Journal. We add, that a Galvanic inftitution for curing difeafes by the Galvanic ftimulus has been announced by a practitioner in the dominions of the Duke of Brunfwick.

